# Cardiometabolic index predicts postoperative atrial fibrillation after isolated CABG: ROC-based comparison with BMI and visceral adiposity indices

**DOI:** 10.17305/bb.2026.13693

**Published:** 2026-02-06

**Authors:** Ercan Kahraman, Şirin Cetin

**Affiliations:** 1 Department of Cardiovascular Surgery, Faculty of Medicine Amasya University, Amasya, Türkiye; 2 Department of Biostatistics, Faculty of Medicine Amasya University, Amasya, Türkiye

**Keywords:** Cardiometabolic index, coronary artery bypass grafting, postoperative atrial fibrillation, visceral adiposity

## Abstract

The assessment of visceral adipose tissue activity has gained significance in cardiac risk stratification. This study evaluates the predictive performance of novel visceral adiposity indices in determining the risk of postoperative atrial fibrillation in patients undergoing isolated coronary artery bypass grafting. Visceral adiposity indices were derived from anthropometric measurements and biochemical parameters collected during the preoperative period. The discriminative abilities of these indices were compared using receiver operating characteristic (ROC) curve analysis and their corresponding area under the curve (AUC) values. Univariate analysis revealed significant associations between the occurrence of postoperative atrial fibrillation and factors such as diabetes mellitus, a high EuroSCORE II, and extended cardiopulmonary bypass duration. Conversely, the visceral adiposity indices demonstrated substantial predictive value for postoperative atrial fibrillation. Notably, the cardiometabolic index (CMI) emerged as a significant predictor for the development of postoperative atrial fibrillation (OR: 4.054, 95% CI: 1.77–9.23; *P* ═ 0.010). These findings indicate that CMI, a composite measure of visceral adiposity and metabolic dysfunction, may provide superior predictive performance for postoperative atrial fibrillation risk following isolated coronary artery bypass grafting compared to body mass index and visceral adiposity index, while showing comparable diagnostic value to the lipid accumulation product and body roundness index. Given the exploratory nature of this study, the suggested cutoff values should be interpreted cautiously and necessitate validation in diverse patient populations and larger cohorts prior to clinical implementation.

## Introduction

Postoperative atrial fibrillation (POAF) is one of the most prevalent arrhythmias following cardiac surgery [1]. It commonly develops after various surgical procedures, including coronary artery bypass grafting (CABG), and is strongly associated with adverse outcomes such as stroke, extended intensive care unit stays, and early postoperative complications [2]. Therefore, predicting the risk of POAF is crucial for enhancing perioperative management strategies.

Obesity is a recognized risk factor for the development of atrial fibrillation [3]. However, the body mass index (BMI), a traditional metric for assessing obesity, has limitations in reflecting the regional distribution and metabolic activity of adipose tissue, as it is calculated solely based on weight and height. Consequently, BMI may be inadequate for predicting adverse events, particularly those following cardiac surgery [4]. Research indicates that among individuals with similar BMI values, cardiovascular risk significantly escalates with increased abdominal fat accumulation [5]. This limitation highlights the need for novel parameters that more accurately assess visceral adiposity and metabolic burden.

In recent years, various composite anthropometric and biochemical indices have been developed to more effectively reflect visceral adiposity and metabolic risk. Indices such as the Visceral Adiposity Index (VAI), Lipid Accumulation Product (LAP), and Cardiometabolic Index (CMI) provide a more precise evaluation of abdominal fat accumulation and metabolic dysfunction, and are proposed as superior alternatives to the conventional BMI [6, 7].

While visceral adiposity indices like VAI, LAP, and CMI are known to correlate with systemic cardiometabolic risk, their clinical utility in predicting the development of POAF after cardiac surgery remains unclear. To address this knowledge gap, our study aimed to evaluate the predictive power of these advanced indices for POAF in an elective CABG population and to compare their effectiveness against the traditional anthropometric measure, BMI.

## Materials and methods

This retrospective observational study was conducted at the cardiac surgery center of Amasya University Hospital, a tertiary care facility, between January 2023 and March 2025. The study was designed as an exploratory analysis to compare the predictive performance of multiple visceral adiposity indices for POAF. Given the hypothesis-generating nature of the study, formal multiplicity adjustment was not applied.

Adult patients diagnosed with coronary artery disease via coronary angiography and subsequently undergoing isolated CABG, following a consensus decision by the cardiology and cardiovascular surgery council, were included in the study.

Patients with the following characteristics were excluded:

● A history of preoperative atrial fibrillation or other rhythm disturbances
● The need for concomitant cardiac surgical procedures (e.g., valve surgery)
● Undergoing off-pump surgery
● A diagnosis of chronic kidney disease or end-stage liver failure
● Requirement for an intra-aortic balloon pump (IABP)
● A left atrial diameter >55 mm or advanced heart failure (New York Heart Association (NYHA) class III–IV)
● A history of hyperthyroidism, pericarditis, active inflammatory disease, or active malignancy
● A history of prior cardiac surgery
● Undergoing emergency surgery

The preoperative comorbidity profile of patients was recorded by reviewing medical records and discharge summaries. In this context, the presence of hypertension was defined by a prior physician diagnosis or the regular use of antihypertensive medication. Diabetes mellitus was confirmed by a physician diagnosis, the use of oral hypoglycemic agents or insulin, or an HbA1c level ≥6.5%, in accordance with American Diabetes Association criteria [8]. Similarly, the diagnosis of chronic obstructive pulmonary disease (COPD) was based on Global Initiative for Chronic Obstructive Lung Disease (GOLD) guidelines, and a history of stroke was defined as a prior stroke resulting in permanent neurological deficit, supported by neuroradiological evidence such as computed tomography or magnetic resonance imaging.

### Laboratory and imaging data

All biochemical and hematological analyses were performed at the central laboratory of a tertiary university hospital following standard procedures. Preoperative blood samples were collected in the morning after a 12-hour fasting period during the surgical preparation phase.

Complete blood counts were obtained using a hematology analyzer (Sysmex XN 1000, Japan). Triglycerides, high-density lipoprotein cholesterol (HDL-C), low-density lipoprotein cholesterol (LDL-C), and total cholesterol levels were measured using an enzymatic colorimetric method with a Beckman Coulter AU 5800 (USA) automated biochemistry analyzer. High-sensitivity C-reactive protein (hs-CRP) levels were analyzed using a nephelometric method (SIEMENS BN II system). Additionally, the estimated glomerular filtration rate (eGFR) was calculated using the Modification of Diet in Renal Disease (MDRD) formula.

Coronary angiographic assessments were performed via a transfemoral approach, and Gensini and SYNTAX scores were calculated based on the severity and distribution of lesions [9, 10]. The angiographic scoring was conducted in a blinded manner by an experienced cardiologist who was independent of the study and unaware of the related data.

### Surgical and clinical procedure

All patients underwent isolated CABG surgery via median sternotomy using the cardiopulmonary bypass technique. Cardiac arrest was induced with antegrade cardioplegia administered under cardiopulmonary bypass. Aortic cross-clamp time and cardiopulmonary bypass duration during the operation were recorded from standard pump record forms completed intraoperatively.

The development of POAF was monitored through continuous electrocardiographic (ECG) monitoring in the intensive care unit. For patients transferred to the hospital ward, daily routine 12-lead ECG results were evaluated. POAF was defined as an arrhythmia lasting at least 30 s, characterized by irregular RR intervals and the absence of P waves, detected via monitoring or a 12-lead ECG within the first 5 postoperative days in patients with no prior history of atrial fibrillation.

The preoperative risk profile of the patients was assessed using European System for Cardiac Operative Risk Evaluation II (EuroSCORE II) values. In the postoperative period, data such as the need for inotropic support and the Vasoactive-Inotropic Score (VIS) were obtained from patient records.

### Anthropometric measurements and index calculations

Preoperative anthropometric measurements were conducted with patients in a standing, relaxed position, wearing light clothing or underwear. Height (cm) was measured using a portable stadiometer, and body weight (kg) was measured with a calibrated digital scale. Waist circumference was measured at the midpoint between the iliac crest and the lower costal margin, while hip circumference was determined at the widest point of the gluteal region using a non-stretchable measuring tape. BMI was calculated by dividing weight (kg) by the square of height (m^2^).

Using preoperative anthropometric and biochemical data, the composite body composition and metabolic risk indices—VAI, Weight-adjusted Waist Index (WWI), LAP, Body Roundness Index (BRI), and CMI—were calculated [11–15].

### VAI:

VAI (Male) ═ [waist circumference (WC) / (39.68 + 1.88 × BMI)] × (TG / 1.03) × (1.31 / HDL)

VAI (Female) ═ [WC / (36.58 + 1.89 × BMI)] × (TG / 0.81) × (1.52 / HDL)

### 2.5 WWI:

WWI = WC / √(weight)

### LAP:

LAP (Male) ═ (WC -- 65) × TG

LAP (Female) ═ (WC -- 58) × TG

### BRI:

BRI = 364.2 -- 365.5 × √[1 -- (WC / 2π)^2^ / (0.5 × height)^2^]

### CMI:

CMI ═ (TG / HDL) × (WC / height)

### Statistical analysis

All statistical analyses were conducted using SPSS version 25.0 software (IBM Corp., Armonk, NY, USA) and the STATA package. The distribution of continuous variables was assessed using the Shapiro-Wilk test. Normally distributed data are presented as mean ± standard deviation, while non-normally distributed data are expressed as median values. Intergroup comparisons were performed using the independent samples *t*-test for normally distributed data and the Mann–Whitney *U* test for non-normally distributed data. Categorical variables are expressed as counts (*n*) and percentages (%); comparisons were made using the chi-square test or, when appropriate, Fisher’s exact test.

The univariate logistic regression analysis was employed to evaluate the explanatory values of clinical and anthropometric variables associated with POAF development. Each adiposity index was analyzed separately to mitigate concerns regarding multicollinearity. To assess the diagnostic performance of each visceral adiposity index in predicting POAF, receiver operating characteristic (ROC) curve analysis was conducted. The overall discriminatory power of the indices was statistically compared by evaluating their Area Under the Curve (AUC) values using the DeLong test. Subsequently, the Youden Index was used to determine the optimal diagnostic threshold for each index, with corresponding sensitivity and specificity values calculated at these cutoff points. Results were reported as odds ratios (ORs) with 95% confidence intervals (CI). A *P*-value of <0.05 was deemed statistically significant for all tests.

### Ethical statement

This retrospective observational study received approval from the Institutional Ethics Committee of Amasya University Faculty of Medicine (Approval Date: 30/06/2025, Approval Number: 2025/120). Due to the retrospective nature of the study and the utilization of anonymized data from medical records, the ethics committee waived the requirement for informed consent. All procedures adhered to the ethical principles outlined in the Declaration of Helsinki.

## Results

### Demographic and clinical characteristics

Between January 2023 and March 2025, a total of 266 patients who underwent CABG surgery were screened for eligibility. Of these, 66 patients were excluded based on the following criteria: concomitant cardiac surgical procedures (*n* ═ 18), off-pump surgery (*n* ═ 13), preoperative atrial fibrillation or rhythm disturbances (*n* ═ 12), emergency surgery (*n* ═ 8), left atrial diameter >55 mm or advanced heart failure (*n* ═ 7), and other exclusion criteria (*n* ═ 8). After applying the exclusion criteria, 200 patients were included in the study. Patients were stratified into two groups based on the development of POAF: those who developed POAF (Group 1, *n* ═ 29, 14.5%) and those who did not (Group 2, *n* ═ 171, 85.5%). The gender distribution was similar between the two groups (male ratio: 82.8% vs. 83.6%; *P* ═ 0.544). Among comorbidities, only diabetes mellitus was significantly associated with POAF (*P* ═ 0.006). No significant differences were observed between the groups regarding hypertension, COPD, and stroke (all *P* > 0.05) (Table 1).

**Table 1:** Comparative analysis of categorical variables across groups

	**Group 1 (*n* ═ 29 14.5%)**	**Group 2 (*n* ═ 171 85.5%)**	***P* value**
Male gender	24 (82.8%)	143 (83.6%)	0.544
History of stroke	0 (0.0%)	2 (1.2 %)	0.642
Presence of hypertension	2 (6.9%)	15 (8.8%)	0.292
Presence of COPD	0 (0.0%)	4 (2.3%)	0.407
Presence of diabetes	15 (51.7%)	35 (20.5%)	**0.006**
Use of inotropes	19 (65.5%)	67 (39.2%)	**0.007**
In-hospital mortality	1 (3.4%)	4 (2.3%)	0.635

Abbreviation: COPD: Chronic obstructive pulmonary disease.

The EuroSCORE II value was significantly higher in patients who developed POAF (*P* ═ 0.021), while the SYNTAX and Gensini scores did not differ significantly between the groups (*P* ═ 0.887 and *P* ═ 0.357, respectively). Additionally, the cross-clamp and cardiopulmonary bypass (CPB) durations were longer in the POAF group. The requirement for inotropic support was also significantly associated with POAF (*P* ═ 0.007). However, mortality rates were comparable in both groups (*P* ═ 0.635) (Table 2).

**Table 2:** Comparative analysis of clinical and laboratory parameters across groups

	**Group 1 (*n* ═ 29) (Mean ± SD) or Median (IQR)**	**Group 2 (*n* ═ 171) (Mean ± SD) or Median (IQR)**	**Confidence interval (95% CI)**	***P* value**
Age (years)	63.62 ± 8.75	61.53 ± 8.82	--1.4–5.58	0.242
Glucose (mg/dL)	161.24 ± 63.71	142.75 ± 38.55	1.45–35.52	**0.033**
BUN (mg/dL)	36.88 ± 18.24	33.36 ± 13.05	--3.9–10.93	0.359
Creatinine (mg/dL)	2.51 ± 6.37	1.60 ± 4.05	--1.48–3.31	0.454
GFR (mL/min/1.73 m^2^)	77.47 ± 18.25	83.29 ± 17.21	--14.92–3.29	0.217
Total cholesterol (mg/dL)	173.24 ± 55.38	192.83 ± 59.20	--51.04–11.86	0.228
Triglycerides (mg/dL)	231.0 (145.6--366.5)	153.7 (102.0--231.3)	23.19–131.96	**<0.001**
LDL-C (mg/dL)	145.43 ± 98.67	124.80 ± 57.02	--5.26–46.52	0.122
HDL-C (mg/dL)	38.79 ± 7.84	42.26 ± 11.81	--8.04–1.09	0.148
AST (U/L)	20.00 ± 6.45	22.78 ± 10.32	--7.85–2.29	0.286
ALT (U/L)	21.17 ± 10.34	29.01 ± 27.73	--21.1–5.41	0.247
CRP (mg/L)	10.1 (5.2--19.4)	6.7 (3.0--14.8)	--2.80–9.70	0.652
WBC (10^3^/µL)	7.86 ± 1.95	7.25 ± 1.68	--0.3–1.52	0.191
Hb (g/dL)	12.83 ± 2.05	13.24 ± 1.69	--1.34–0.51	0.374
Hct (%)	39.11 ± 5.55	40.91 ± 11.25	--7.24–3.65	0.517
Platelet (10^3^/µL)	243.17 ± 62.15	229.52 ± 77.02	--25.26–52.56	0.492
Neutrophils (10^3^/µL)	4.78 ± 1.47	4.43 ± 1.66	--0.5–1.2	0.413
Lymphocytes (10^3^/µL)	2.16 ± 0.95	2.09 ± 0.67	--0.31–0.46	0.76
HbA1c (%)	7.36 ± 2.06	7.05 ± 1.92	--0.71–1.33	0.559
VIS	348.11 ± 542.28	158.23 ± 468.27	--62.83–442.59	0.141
Intubation time (h)	8.72 ± 3.14	8.68 ± 4.54	--2.22–2.29	0.973
ICU stay (days)	4.29 ± 3.33	2.66 ± 2.11	0.69–2.55	**<0.001**
Gensini score	90.88 ± 38.60	76.88 ± 30.63	--43.82–15.82	0.357
SYNTAX score	22.25 ± 6.04	22.56 ± 8.19	--4.4–3.78	0.887
EuroSCORE II	3.90 ± 2.81	2.90 ± 2.00	0.15–1.84	**0.021**
Cross-clamp time (min)	75.24 ± 28.78	65.50 ± 23.96	--0.04–19.52	**0.045**
CPB time (min)	126.21 ± 38.17	104.34 ± 32.46	8.67–35.06	**<0.001**

Abbreviations: ALT: Alanine aminotransferase; AST: Aspartate aminotransferase; BUN: Blood urea nitrogen; CPB time: Cardiopulmonary bypass time; CRP: C-reactive protein; Creatinine: Serum creatinine; GFR: Glomerular filtration rate; Glucose: Fasting blood glucose; Gensini score: Gensini score; Hb: Hemoglobin; Hct: Hematocrit; HDL-C: High-density lipoprotein cholesterol; ICU stay: Length of intensive care unit stay; LDL-C: Low-density lipoprotein cholesterol; VIS: Vasoactive-inotropic score; WBC: White blood cell count.

### Anthropometric, biochemical, and hematological parameters

The mean age was similar between the groups (*P* ═ 0.242). Although BMI values trended higher in the POAF group, this difference did not achieve statistical significance (29.6 ± 4.5 vs. 27.6 ± 6.4 kg/m^2^; *P* ═ 0.112). In contrast, waist circumference (*P* ═ 0.012) and triglyceride levels (*P* < 0.001) were significantly higher in the POAF group. All indices reflecting visceral adiposity (VAI, LAP, CMI, and BRI) were statistically significantly higher in the POAF group (*P* ═ .011 for LAP; *P* < .001 for all other indices) (Table 3).

**Table 3:** Comparative analysis of anthropometric indices across groups

	**Group 1 (Mean ± SD)**	**Group 2 (Mean ± SD)**	**Confidence interval (95% CI)**	***P* value**
Height (cm)	165.72 ± 8.71	168.11 ± 8.28	--5.69--0.92	0.161
Weight (kg)	81.10 ± 14.15	77.07 ± 13.31	--1.28--9.36	0.144
BMI	29.56 ± 4.50	27.57 ± 6.41	--0.46--4.43	0.112
Waist circumference (cm)	108.73 ± 16.83	101.55 ± 13.05	1.52--12.83	**0.012**
Hip circumference (cm)	105.13 ± 8.82	102.31 ± 9.17	--2.34--7.98	0.289
VAI	14.03 ± 16.60	7.48 ± 6.65	3.07--10.02	**<0.001**
WWI	9.89 ± 11.31	7.99 ± 8.72	--5.52--1.71	0.301
LAP	94.71 ± 61.38	70.75 ± 43.72	--42.43 to --5.50	**0.011**
BRI	7.08 ± 2.58	5.66 ± 1.74	0.64--2.20	**<0.001**
CMI	2.50 ± 2.93	1.31 ± 1.14	0.58--1.81	**<0.001**

Abbreviations: BMI: Body mass index; BRI: Body roundness index; CMI: Cardiometabolic index; LAP: Lipid accumulation product; VAI: Visceral adiposity index; WWI: Weight-adjusted waist index.

**Figure 1.**
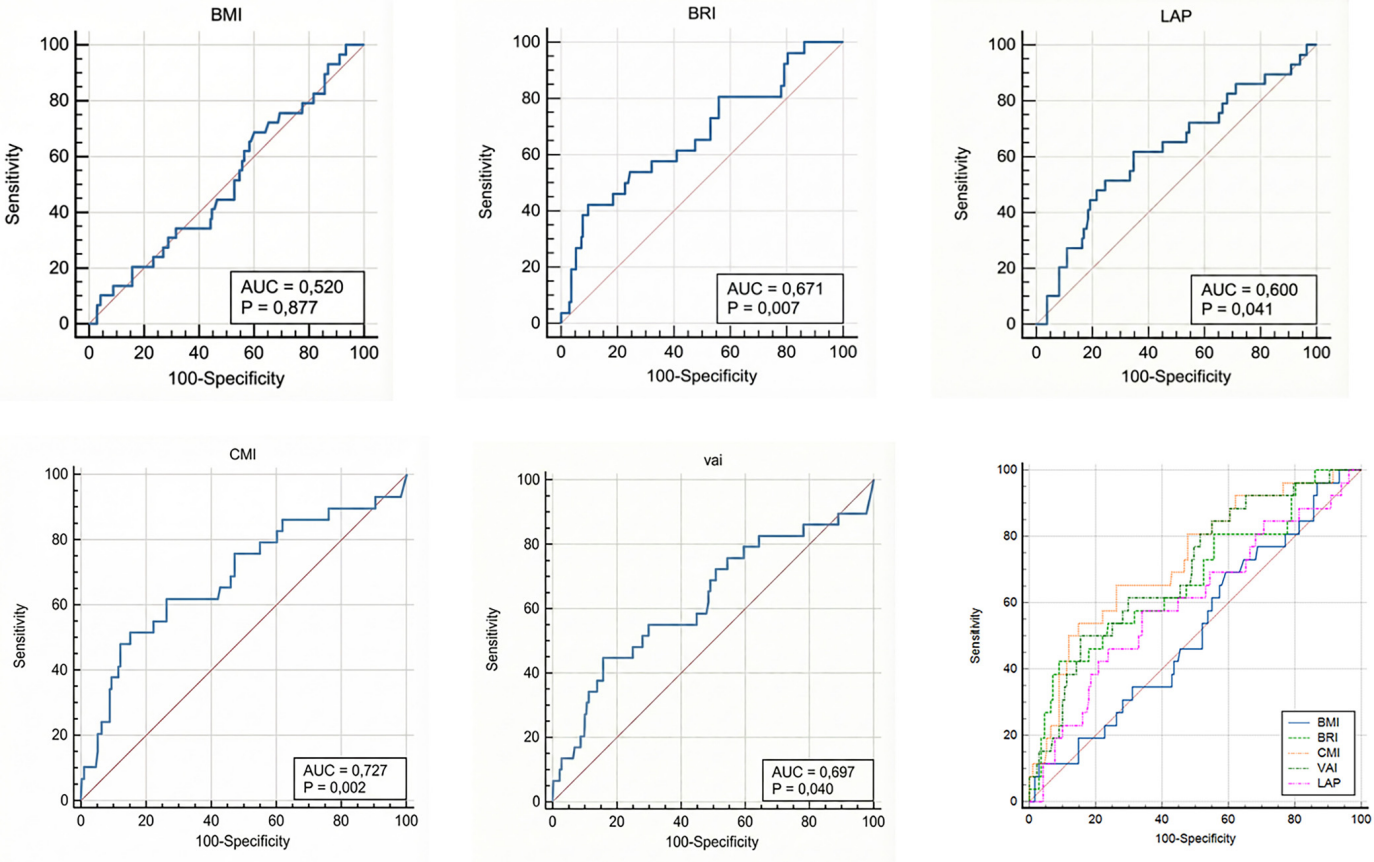
**ROC curves comparing the discriminatory performance of preoperative anthropometric and composite visceral adiposity indices for predicting POAF after isolated CABG**. Individual panels display ROC curves for BMI, BRI, LAP, CMI, and VAI, and the lower panel overlays all curves to facilitate direct visual comparison. For each index, the AUC and the corresponding *P* value (testing AUC against 0.5) are shown within the panel; the diagonal line indicates the no-discrimination reference. CMI demonstrated the highest discrimination (AUC = 0.727; *P* ═ 0.002), followed by VAI (AUC = 0.697; *P* ═ 0.040) and BRI (AUC = 0.671; *P* ═ 0.007), whereas LAP showed modest discrimination (AUC = 0.600; *P* ═ 0.041). BMI exhibited no significant discriminatory ability (AUC = 0.520; *P* ═ 0.877). Abbreviations: AUC: Area under the curve; BMI: Body mass index; BRI: Body roundness index; CABG: Coronary artery bypass grafting; CMI: Cardiometabolic index; LAP: Lipid accumulation product; POAF: Postoperative atrial fibrillation; ROC: Receiver operating characteristic; VAI: Visceral adiposity index.

### Diagnostic performance and predictive values of indices

ROC curve analyses were conducted to evaluate the diagnostic performance of visceral adiposity indices for predicting POAF (Figure 1). At optimal cutoff values determined by the Youden Index, CMI (>1.89) exhibited the most favorable diagnostic profile, achieving the highest Youden Index (0.365) with a sensitivity of 51.7% and a specificity of 84.8% (AUC = 0.727). Among the other indices, BRI was notable for its particularly high specificity (90.5%), while LAP provided the highest sensitivity (62.1%). In contrast, BMI demonstrated poor discriminatory ability that did not reach statistical significance (Table 4).

**Table 4:** Diagnostic performance of anthropometric indices in predicting POAF

	**AUC**	**95% CI**	***P*-value**	**Youden index**	**Cut-off**	**Sensitivity (%)**	**Specificity (%)**
BMI	0.520	0.447–0.592	0.877	0.096	≥26.20	34.5	55.9
BRI	0.671	0.600–0.737	**0.006**	0.328	>7.52	42.3	90.5
VAI	0.697	0.627–0.761	**0.039**	0.290	>11.08	44.8	84.2
LAP	0.600	0.527–0.669	**0.041**	0.270	≥53.74	62.1	64.9
CMI	0.727	0.658–0.788	**0.002**	0.365	>1.89	51.7	84.8

Abbreviations: BMI: Body mass index; BRI: Body roundness index; CMI: Cardiometabolic index; LAP: Lipid accumulation product; VAI: Visceral adiposity index.

Comparative analyses using the DeLong test revealed that CMI and VAI had statistically superior discriminative ability compared to BMI (ΔAUC = 0.207, *P* ═ 0.009 and ΔAUC = 0.177, *P* ═ 0.028, respectively), whereas the differences between BMI and the other indices did not achieve statistical significance (Table 5).

**Table 5:** Comparative analysis of predictive performance among anthropometric indices

**Comparison**	**Difference in AUC (Second-first)**	**95% CI**	***P*-value**
BMI - BRI	0.151	--0.024--0.326	0.090
BMI - VAI	0.177	0.019--0.336	**0.028**
BMI - LAP	0.080	--0.105--0.265	0.395
BMI - CMI	0.207	0.050--0.363	**0.009**
BRI - VAI	0.026	--0.102--0.154	0.688
BRI - LAP	--0.071	--0.108--0.251	0.436
BRI - CMI	0.055	--0.066--0.178	0.370
VAI - LAP	--0.097	--0.098--0.293	0.329
VAI - CMI	0.029	0.009--0.050	**0.004**
LAP - CMI	0.127	--0.064--0.319	0.194

Abbreviations: BMI: Body mass index; BRI: Body roundness index; CMI: Cardiometabolic index; LAP: Lipid accumulation product; VAI: Visceral adiposity index.

**Table 6:** Predictive value of body composition indices for postoperative atrial fibrillation risk

**Cut off value**	**Odds ratio**	**(95% CI)**	***P* value**
BRI> 7.52	5.536	2.46–13.64	***P* < 0.001**
BMI≥ 26.2	1.909	0.80–4.55	0.145
VAI> 11.08	3.978	1.72–7.95	**0.010**
WWI>11.07	1.34	0.61–2.96	0.460
LAP≥ 53.74	4.018	1.77–9.08	**0.010**
CMI> 1.89	4.054	1.77–9.23	**0.010**

Abbreviations: BMI: Body mass index; BRI: Body roundness index; CMI: Cardiometabolic index; LAP: Lipid accumulation product; VAI: Visceral adiposity index; WWI: Weight-adjusted waist index.

Logistic regression analysis confirmed the predictive value of visceral adiposity indices for POAF. With the exception of the WWI (*P* ═ 0.460), all other novel visceral adiposity indices (BRI, VAI, LAP, CMI) demonstrated significant associations with POAF risk, with odds ratios ranging from approximately four to five-fold. Notably, CMI (>1.89) was associated with more than a four-fold increased risk for POAF (OR = 4.054, 95% CI: 1.77–9.23; *P* ═ 0.010). Conversely, BMI, a traditional measure, failed to demonstrate a statistically significant predictive value (Table 6).

## Discussion

POAF, a common complication following cardiac surgery, contributes to severe adverse clinical outcomes, including stroke and prolonged intensive care unit stays. Visceral adiposity, implicated in the pathophysiology of atrial fibrillation, emerges as a significant risk factor in this context. The primary finding of this study indicates that a novel index reflecting visceral adiposity may serve as a valuable predictor for POAF. In contrast to BMI, a traditional metric, CMI has emerged as a promising marker with the highest diagnostic performance for potential clinical application.

### Role of visceral adiposity in the pathophysiology of POAF

It is widely accepted that visceral adipose tissue functions not merely as a passive energy reservoir but as a metabolically active endocrine organ [16]. This tissue releases various proinflammatory cytokines and adipokines into the systemic circulation, resulting in a state of low-grade chronic inflammation [17]. Previous studies have established that increased visceral adipose tissue, particularly when quantified by gold-standard methods such as computed tomography, is an independent risk factor for atrial fibrillation in the general population [18–20]. The underlying mechanisms for this association are proposed to include systemic inflammation, oxidative stress, autonomic dysfunction, and subsequent atrial remodeling [21].

Given this evidence-based relationship, accurate quantification of visceral adiposity is essential for effective risk assessment in clinical practice. However, the applicability of gold-standard imaging modalities, such as computed tomography or magnetic resonance imaging, is limited in routine screening due to factors such as cost and potential radiation exposure. This limitation necessitates the identification of simpler, cost-effective, and accessible markers that indirectly reflect visceral adiposity. Among the parameters used for this purpose, BMI has been the most commonly preferred metric.

### Insufficiency of BMI in predicting POAF

The fundamental limitation of BMI lies in its inability to differentiate between fat mass and lean mass, as well as its failure to provide information regarding the regional distribution of body fat [22]. This situation has led to contradictory findings, known as the “obesity paradox,” particularly in cardiovascular risk assessment, thereby questioning the reliability of this metric [23]. Similar inconsistencies exist specifically in the context of atrial fibrillation; while some studies have identified BMI as a risk factor, many have failed to demonstrate a significant association between BMI and the development of atrial fibrillation [24–26].

Our study clarifies the reasons for inconsistencies in the literature regarding the inadequacy of BMI in assessing the risk of POAF. Our findings indicate no significant difference in BMI between patients who developed POAF and those who did not (*P* = 0.112). This conclusion is further supported by ROC analysis, which yielded nearly identical reference lines. These observations suggest that BMI inadequately reflects the proarrhythmic risk associated with visceral adipose tissue accumulation, thus limiting its predictive capacity for POAF.

### Superiority of novel visceral adiposity indices

In light of the structural limitations of BMI, novel composite indices such as VAI, LAP, and CMI have been developed to provide a more comprehensive assessment of visceral adiposity and its metabolic consequences by integrating anthropometric measures with metabolic markers. The literature consistently reports the superiority of these composite indices over BMI in predicting various cardiometabolic conditions, including diabetes, hypertension, and metabolic syndrome [11, 15]. This has prompted the hypothesis that these indices may also hold prognostic value for other cardiovascular complications, such as POAF. Recent evidence indicates that VAI and LAP can predict the risk of incident atrial fibrillation (AF) in the general population [27, 28]. However, whether these indices possess unique prognostic value independent of established risk factors remains an open question.

### Validation of established risk factors

Although our primary focus was on visceral adiposity indices, our analyses also allowed for the evaluation of well-established risk factors for POAF reported in the literature. Our results demonstrated that the prevalence of diabetes, EuroSCORE II values, and cardiopulmonary bypass durations were significantly higher in the POAF group. These findings align with existing evidence identifying diabetes, increased surgical risk scores, and prolonged perfusion times as independent risk factors for POAF [29–31]. This consistency suggests that our study cohort is representative of the general patient population undergoing isolated CABG surgery, supporting the external validity of our findings. It is important to note that perioperative factors such as cardiopulmonary bypass duration and inotrope requirements cannot be utilized for preoperative risk stratification. However, since POAF typically develops between postoperative days 2–5, these variables remain clinically useful for early risk assessment upon intensive care unit (ICU) admission. In contrast, visceral adiposity indices facilitate risk stratification prior to surgery.

### Diagnostic performance and potential advantages of CMI

Following the identification of significant associations between visceral adiposity indices and POAF, we compared the diagnostic performance of these indices to determine which marker exhibited the most favorable characteristics for clinical application. Our analyses revealed that both CMI and VAI demonstrated statistically superior discriminative abilities compared to BMI (Table 5), while the novel visceral adiposity indices (CMI, VAI, BRI, LAP) showed significant predictive value for POAF, in contrast to BMI, which did not reach statistical significance (Table 6). This overall finding indicates that BMI fails to capture the role of visceral obesity in the pathogenesis of POAF.

For VAI (>11.08), we observed an approximately fourfold increased risk (OR=3.978) and an AUC of 0.697, consistent with previous studies identifying VAI as a risk factor in general AF populations [27]. Notably, the higher AUC in our cohort suggests that this index may have pronounced prognostic value in patients experiencing acute surgical stress.

The most striking finding of our study is that it not only demonstrates the superiority of next-generation indices over BMI but also identifies CMI as having the highest numeric AUC value among the evaluated indices. While CMI exhibited statistically superior discriminative ability compared to BMI and VAI, its performance did not significantly differ from that of LAP or BRI. CMI demonstrated the highest diagnostic accuracy (AUC=0.727) and the most optimal balance (Youden Index=0.365; sensitivity 51.7%, specificity 84.8%) and indicated that a threshold >1.89 increases POAF risk more than fourfold (OR=4.054).

The CMI formula incorporates two fundamental components of POAF pathophysiology, which may account for its predictive performance. The first component, the Triglyceride/HDL ratio, is recognized as one of the strongest markers of insulin resistance and atherogenic dyslipidemia [32]. This ratio reflects not only lipid levels but also endothelial dysfunction and systemic inflammation [33]. The second component, the Waist-to-Height ratio, emphasizes fat distribution in the body, offering a more precise quantification of central obesity compared to BMI. Consequently, CMI integrates both the metabolic activity of visceral fat and the anatomical risk associated with its accumulation into a single formula. This holistic approach may explain why CMI exhibited the most favorable diagnostic performance among the evaluated indices.

Emerging evidence suggests that anatomical parameters may also contribute to atrial fibrillation risk stratification. The Modified Haller Index (MHI), which reflects chest wall conformation, has been inversely associated with asymptomatic status in persistent atrial fibrillation patients, with lower MHI values indicating a higher likelihood of underlying structural cardiomyopathy and left atrial dysfunction [34]. While MHI addresses anatomical dimensions rather than metabolic dysfunction, its integration with visceral adiposity indices such as CMI may provide a more comprehensive approach to cardiovascular risk stratification in future studies.

### Strengths and limitations of the study

This study has several strengths that contribute significantly to the literature. To the best of our knowledge, it is the first to comprehensively compare the performance of a range of novel visceral adiposity indices, including VAI, LAP, CMI, and BRI, in predicting POAF among patients undergoing isolated CABG. Additionally, all indices examined can be easily calculated from routine preoperative clinical and laboratory data, enhancing their practical applicability in clinical settings.

However, our study has limitations that warrant consideration. The retrospective and single-center design may limit the generalizability of our findings to other patient populations or surgical protocols. Furthermore, some perioperative variables that could affect the risk of POAF, such as antiarrhythmic prophylaxis measures and electrolyte status, were not systematically recorded in our dataset. The optimal threshold values were derived and evaluated using the same dataset, suggesting that these findings should be viewed as exploratory rather than definitive. External validation in prospective studies involving independent cohorts and additional perioperative parameters will be necessary to enhance the clinical applicability of these indices.

## Conclusion

This exploratory study suggests that, in patients undergoing isolated CABG, CMI offers better predictive performance for assessing visceral adiposity and metabolic dysfunction compared to BMI and VAI. CMI demonstrated the highest predictive value among the evaluated indices for POAF risk, facilitating a more precise and holistic assessment of the metabolic profile. These preliminary findings indicate that CMI may serve as a practical biomarker in clinical settings for the early identification of patients with metabolic syndrome, obesity, and cardiometabolic risk, enabling targeted preventive and therapeutic strategies. Given that the cutoff values were derived and tested within the same dataset, external validation in prospective studies is needed to confirm their clinical utility. The potential advantages of CMI warrant further validation in studies involving diverse patient populations and larger sample sizes.

## Data Availability

The data supporting the findings of this study are available from the corresponding author upon reasonable request.
